# Tanshinone I inhibits the growth and metastasis of osteosarcoma via suppressing JAK/STAT3 signalling pathway

**DOI:** 10.1111/jcmm.14539

**Published:** 2019-07-10

**Authors:** Weiguo Wang, Jinsong Li, Zhiyu Ding, Yuezhan Li, Jianlong Wang, Shijie Chen, Jinglei Miao

**Affiliations:** ^1^ Department of Orthopaedics The Third Xiangya Hospital of Central South University Changsha China

**Keywords:** JAK/STAT3 signalling pathway, osteosarcoma, tanshinone I, tumour growth and metastasis

## Abstract

Tanshinone I (Tan I) is a widely used diterpene compound derived from the traditional Chinese herb Danshen. Increasing evidence suggests that it exhibits anti‐cancer activity in various human cancers. However, the in vitro and in vivo effects of Tan I on osteosarcoma (OS) remain inadequately elucidated, especially those against tumour metastasis. Our results showed that Tan I significantly inhibited OS cancer cell proliferation, migration and invasion and induced cell apoptosis in vitro. Moreover, treatment with 10 and 20 mg/kg Tan I effectively suppressed tumour growth in subcutaneous xenografts and orthotopic xenograft mouse models. In addition, Tan I significantly inhibited tumour metastasis in intracardiac inoculation xenograft models. The results also showed that Tan I‐induced increased expression of the proapoptotic gene Bax and decreased expression of the anti‐apoptotic gene Bcl‐2 is the possible mechanism of its anti‐cancer effects. Tan I was also found to abolish the IL‐6‐mediated activation of the JAK/STAT3 signalling pathway. Conclusively, this study is the first to show that Tan I suppresses OS growth and metastasis in vitro and in vivo, suggesting it may be a potential novel and efficient drug candidate for the treatment of OS progression.

## 1 INTRODUCTION

Osteosarcoma (OS), classified as an orphan disease, is the most common malignant bone tumour in children and young adults.^1^, ^2^, ^3^, ^4^ With the significant clinical advances over the last decade, current therapies now incorporating surgical resection and combinational chemotherapy could cure 65%‐70% of patients with localized osteosarcoma.^5^, ^6^ However, the 5‐year survival rate for metastatic or relapsed osteosarcoma is still only approximately 20%,^7^, ^8^ highlighting the urgent need for more effective therapies, especially for metastatic OS. Among the emerging novel treatments, traditional Chines medicines, containing multiple bioactive components with specific biological activities, have been widely accepted as promising therapeutic or preventive regimens for malignant diseases.^9^, ^10^ With the advantage of less adverse effects, traditional Chinese medicines could be potential and safe candidates for the prevention and treatment of various cancers such as lung and gastric cancers, as well as OS.^11^, ^12^, ^13^

Tanshinone I (Tan I), one of the most abundant diterpene compounds from the traditional Chinese herb *Salvia miltiorrhiza Bunge* (Danshen), has been approved for clinical treatment of coronary heart and cerebrovascular diseases.^14^, ^15^ Danshen, and especially its derivative tanshinone II A, reportedly performs comprehensive in vitro and in vivo activities against various human cancer cells, including OS.^16^, ^17^, ^18^, ^19^, ^20^, ^21^ Recently, the anti‐cancer effects of Tan I in malignant cancers have also been elucidated. Substantial evidence indicates that Tan I induces apoptosis in myeloid leukaemia, colon, lung, prostate and gastric cancer cells in vitro by activating caspase‐3.^22^, ^23^, ^24^, ^25^, ^26^, ^27^ Moreover, it reportedly suppresses cell growth by down‐regulating adhesion molecules in breast cancer cells,^28^ inhibiting vascular endothelial growth factor (VEGF) expression in lung cancer cells^29^ and decreasing angiogenesis.^30^ A small but growing number of researches have also suggested that Tan I could effectively inhibit lung and prostate cancer growth in vivo.^31^, ^32^, ^33^ Nevertheless, its in vitro and in vitro effects on OS are inadequately elucidated, especially those against OS metastasis.

In this study, we therefore determined the effects of Tan I on the proliferation, apoptosis, migration and invasion of OS cell lines in vitro, and on tumour growth and metastasis in vivo, using xenografts and orthotopic implantation tumour models. We also analysed cellular and molecular biomarkers associated with its functions. For instance, Bcl‐2/Bax is related to apoptosis, and matrix metalloproteinase (MMP)‐2/MMP‐9 is involved in migration and invasion. Moreover, we identified the JAK1/2‐STAT3 signalling pathway as an anti‐metastasis target of Tan I. These results imply the potential of Tan as a novel OS preventive and therapeutic agent.

## 2 MATERIALS AND METHODS

### 2.1 Cell lines, animals, and reagents

The human OS cell lines U2OS and MOS‐J, and the mouse OS cell line OS‐1 were obtained from ATCC. Stable U2OS‐luc and OS‐1‐luc cell lines were established by transfection with the pGL4 vector (Promega) and selection with G418 (Sigma). The U2OS and MOS‐J cells were cultured in Dulbecco's Modified Eagle Medium (Gibco), while OS‐1 cells were maintained in RPMI‐1640 medium (Gibco). The mediums were supplemented with 10% foetal bovine serum (Wisent); then, the cells were incubated in a humidified incubator with 5% CO_2_ at 37℃.

Mice were obtained from the Shanghai branch of China's National Rodent Laboratory Animal Resources. All animal experimental protocols were approved by the Animal Investigation Committee of the Institute of Biomedical Sciences, Central South University.

Tan I was purchased from Sigma, and a 50 mmol/L stock solution was prepared in dimethyl sulfoxide (DMSO; Sigma) and stored at −20°C. Different concentrations of Tan I were prepared by defined dilutions in culture medium. Most appropriate antibodies were purchased from Cell Signaling Technology, unless otherwise stated. Antibodies were used according to the manufacturer's instructions.

### 2.2 Cell viability assay

Cell proliferation was determined using the sulforhodamine B assay.^34^ Briefly, cells were seeded in 96‐well plates at a density of 4 × 10^3^ cells/well for 24 hours and then exposed to different Tan I concentrations for 72 hours. Then, the cells were fixed with 10% trichloroacetic acid for 1 hours at 4℃ and stained with 50 μL of 0.4% (w/v) SRB (Sigma) for 20 minutes at room temperature. Excess dye was removed by repeated washing with 1% (vol/vol) acetic acid, and 100 μL of 10 mmol/L Tris buffer was added for OD determination at 515 nm, using a microplate reader. All experiments were performed in triplicate.

### 2.3 Colony formation assay

U2OS and MOS‐J were seeded at a density of 2 × 10^3^ and 4 × 10^3^ cells/well, respectively, in 6‐well plates for 24 hours recovery and then treated with different Tan I concentrations for a week. The cells were then fixed with 4% paraformaldehyde for 20 minutes at room temperature and stained with 0.2% crystal violet. The number of cell colonies was determined as the ratio of the number of treated to untreated samples. All experiments were performed in triplicate.

### 2.4 Live/dead staining assay

The live/dead assay was conducted with the live/dead viability/cytotoxicity kit (Molecular Probes), following the manufacturer's instructions. Briefly, cells were exposed to different Tan I concentrations for 24 hours. Then, Calcein‐AM (living cell staining, green) and ethidium homodimer‐1 (dead cell staining, red) were added. The green living and red dead cells were visualized using fluorescence microscopy and photographed, and the ratios of dead cells to total cells were calculated.

### 2.5 Migration assay

The cell migration assay was conducted by Boyden inserts (8 μm; BD Biosciences) as previously described.^35^ In brief, serum‐starved U2OS and MOS‐J cells in a medium containing 0.5% FBS were pre‐treated with various Tan I concentrations (0, 0.08, 0.4 and 2.0 μmol/L) for 30 minutes. Then, the cells were seeded at a density of 1.5 × 10^4^ cells/well in the upper chamber of a Transwell plate and allowed to migrate to the lower chamber. After incubation for 24 hours, non‐migrated cells were removed while migrated cells were fixed with 4% paraformaldehyde and stained with 1% crystal violet. The cells were then visualized and photographed with an inverted microscope (Olympus; magnification, ×100). The migrated cells in four random fields were quantified and manually counted, and the percentage of inhibition was expressed using 100% as that of the control well.

### 2.6 Invasion assay

Cell invasion was determined using the wound‐healing migration assay. Briefly, U2OS and MOS‐J cells were seeded in 6‐well plates. After growing to confluent monolayers, “wounds” were carefully created on the cells using a sterile pipette tip. After sounding and washing, the cells were cultured in complete medium with various Tan I concentrations, incubated for 24 hours, fixed with 3.7% paraformaldehyde and photographed at ×100 magnification under a phase‐contrast microscope. Migrated cells were manually quantified, and the percentage of inhibition of migrated cells was expressed using 100% as that of the untreated group.

### 2.7 Apoptosis assay

Cell apoptosis was measured with the Apoptosis Detection Kit (BD Biosciences) using flow cytometry (FACSCalibur; BD Biosciences), per the manufacturer's instructions. The cells were treated with different concentrations of Tan I for 48 hours, harvested using 0.25% trypsin and washed with PBS. Then, they were resuspended and incubated with 5 μL of Annexin V fluorescein isothiocyanate and 5 μL of propidium iodide for 15 minutes in the dark at room temperature. After staining, the cells were resuspended with binding buffer and immediately analysed with flow cytometry.

### 2.8 Quantitative real‐time PCR

Cells were cultured and treated with different concentrations of Tan I for 24 or 48 hours. After cell harvested, total RNA was extracted using TRIzol reagent (Takara), according to the manufacturer's instructions. cDNA was synthesized from RNA with a cDNA reverse transcription kit (Takara). Real‐time PCR was performed in triplicate using gene‐specific primers (Thermo Fisher Scientific), on the Stratagene Mx3005P PCR system (Agilent Technologies). mRNA levels were normalized to β‐actin levels. The gene‐specific primers used are listed in Table 1 and were checked for specificity before use.

**Table 1:** qRT‐PCR primer sets

Gene	Primer set	OMIM ID
Bcl‐2		
Forward	5′‐CGCCCTGTGGATGACTGAGTA‐3′	151430
Reverse	5′‐GGGCCGTACAGTTCCACAAAG‐3′	
Bax		
Forward	5′‐CCCTTTTGCTTCAGGGTTTCATCCA‐3′	600040
Reverse	5′‐CTTGAGACACTCGCTCAGCTTCTTG‐3′	
MMP‐2		
Forward	5′‐CTCAGATCCGTGGTGAGATCT‐3′	120360
Reverse	5′‐CTTTGGTTCTCCAGCTTCAGG‐3′	
MMP‐9		
Forward	5′‐AAGTGGCACCACCACAACAT‐3′	120361
Reverse	5′‐TTTCCCATCAGCATTGCCGT‐3′	
β‐actin		
Forward	5′‐CTGGAGCATGCCCGTATTTA‐3′	102630
Reverse	5′‐TTTGGTCTTGCCACTTTTCC‐3′	
MMP2 Promoter		
Forward	5′‐ATTGGCAGGCCCATTTGGGTTGAT‐3′	
Reverse	5′‐TCAGGGATTCACGGTTGTCACCTT‐3′	
Bcl‐2 Promoter		
Forward	5′‐AGTTCGTGGGGTCATGTGT‐3′	
Reverse	5′‐CCAGGAGAAATCAAACAGAGGC‐3′	

Abbreviations: MMP, matrix metalloproteinase; OMIM, Online Mendelian Inheritance in Man

### 2.9 Chromatin immunoprecipitation (ChIP) assay

ChIP assay was performed to evaluate the interaction between STAT3 and Bcl2 or MMP2, as previously described.^36^ Briefly, cells were treated with Tan I for 24 hours, fixed with 1% formaldehyde to crosslink chromatin and protein, immunoprecipitated with STAT3 or IgG antibodies and then incubated with protein A/G agarose beads. After washing severally, the protein‐DNA complex was reversed by proteinase K treatment, and DNA was recovered and isolated with phenol‐chloroform. The DNA was analysed using qPCR with primers that are specific to regions spanning the Stat3‐binding sites of the Bcl‐2 and MMP2 promoters, listed in Table 1.

### 2.10 Western blotting

Cells were exposed to various concentrations of Tan I for indicated time and lysed in RIPA buffer [50 mmol/L Tris‐HCl (pH 7.4), 150 mmol/L NaCl, 5 mmol/L EDTA, 1% Triton X‐100, 1% sodium deoxycholic acid, 0.1% SDS, 2 mmol/L phenylmethylsulfonyl fluoride (PMSF), 30 mmol/L Na_2_HPO_4_, 50 mmol/L NaF, 1 mmol/L Na_3_VO_4_] containing protease/phosphatase inhibitors (Roche). Lysates were denatured with SDS loading buffer and heated at 100℃ for 5 minutes. After fractionation with SDS‐polyacrylamide gel electrophoresis (PAGE), proteins were transferred to nitrocellulose membranes. The membranes were blocked with 5% skim milk in PBS and 0.1% Tween‐20 and then probed with specific primary antibodies overnight at 4℃. The membranes were then incubated with secondary antibodies for 2 hours at room temperature. The blots were visualized using the Odyssey fluorescence scanner (LI‐COR Bioscience Inc). The antibodies used were Bax (2772, 1:1000), Bcl‐2 (3498, 1:1000), MMP‐9 (13667, 1:1000), MMP‐2 (87809, 1:1000), JAK1 (3344, 1:1000), P‐JAK1 (74129, 1:1000), JAK2 (3230, 1:1000), P‐JAK2 (3771, 1:1000), STAT3 (12640, 1:1000), P‐STAT3 (9145, 1:1000) and β‐actin (4970, 1:1000) from Cell Signaling Technology.

### 2.11 Haematoxylin and eosin (H&E) staining

Haematoxylin and eosin staining was performed as previously described.^37^ Tumour or mouse tissue samples were immediately fixed in 10% neutral buffered formaldehyde for 24 hours, progressively dehydrated in solutions containing increasing percentages of ethanol (75, 85, 95 and 100%, vol/vol) and embedded in paraffin. Then, the paraffin‐embedded sections (2.5 μm) were treated with xylene I for 10 minutes, xylene II for 10 minutes, a 1:1 mixture of anhydrous ethanol and xylene, and ethanol for 10 minutes, for dewaxing and hydration. Next, the sections were stained (nucleus) using haematoxylin for 10 minutes and washed twice with ethanol containing 0.25% hydrochloric acid. Then, the sections were stained (cytoplasm) with 0.5% ethanol containing eosin for 1 minute and dehydrated with ethanol for 20 minutes. The sections were later treated with solutions containing increasing percentages of ethanol and xylene I and II for 5 minutes and mounted in neutral balsam. The sections were visualized and photographed using a phase‐contrast microscope. Ten fields in each section were randomly chosen for quantification and analyses.

### 2.12 Immunohistochemistry (IHC)

Immunohistochemistry analysis was performed as previously reported.^37^ Briefly, tumour samples were immediately fixed in 4% formaldehyde for 24 hours, progressively dehydrated in solutions containing increasing percentages of ethanol (75, 85, 95 and 100%, v/v), embedded in paraffin and cut into 4 μm sections. The sections were stained with anti‐Bax (1:1000) and anti‐MMP9 (1:1000) as primary antibodies, and HRP‐labelled secondary antibodies (1:1000). After diaminobenzidine (DAB) staining, the sections were both photographed and analysed under an optical microscope.

### 2.13 Xenograft model of human osteosarcoma cancer U2OS tumour

As previously described, 38 U2OS cells (2 × 10^6^) were subcutaneously inoculated on the right side of the dorsal area of 4‐week‐old male nude mice. After the tumours reached about 100 mm^3^, the mice were randomly divided into 3 groups (n = 5). Then, Tan I (10 or 20 mg/kg/d) was intraperitoneally injected into each mouse for 20 days. The control group was treated with dimethyl sulfoxide. During the administration period, the bodyweights and tumour sizes of the mice were monitored every 2 days. Twenty days from treatment commencement, the mice were killed, and their tumours were harvested, weighed and photographed. The growth rate of the tumour xenografts was evaluated by determining the tumour volume using a digital calliper. The tumour volume was calculated using the equation volume (V) = length × width^2^ × 0.52.

### 2.14 OS‐1‐luc orthotopic transplantation xenograft model

Orthotopic implantation of tumour cells was performed as previously described.^39^ Briefly, C57 mice were randomly assigned to separate cages (4 mice/cage) for each experiment, and all procedures were performed in a sterile environment. Mice were anesthetized with xylazine and ketamine, and 2 × 10^5^ OS‐1‐luc cells suspended in 10 μL of sterile PBS were orthotopically injected into their left tibiae using tuberculin syringes with 29‐gauge needles. Buprenorphine (0.075 mg/kg; Buprenex^®^; Reckitt Benckiser Healthcare) was intraperitoneally injected every 8 hours for pain control over the first 24 hours post‐tumour cell implantation. Two days later, mice bearing tumour were randomly divided into 3 groups, according to photon flux indexes detected by Xenogen IVIS 2000 Luminal Imager. Then, Tan I (10 or 20 mg/kg/d) was intraperitoneally injected into the mice for 21 days. Tumour growth was monitored by measuring the width (W) and length (L) of the proximal tibia and the stifle joint weekly, using digital callipers and in vivo imaging as described. Bioluminescence imaging was conducted after injection of D‐luciferin (Gold Biotechnology), following isoflurane inhalant anaesthesia, and analysed with the Living Image software (Calliper Life Sciences). The growth rate curves of the tumour xenografts were established using the photon flux indexes. The bone tissue volume (V) of each mouse was calculated from their tibiae using the equation V = length × width^2^ × 0.52, and tumour volume was estimated using the equation V = volume of the affected (left) tibia − volume of the contralateral unaffected (right) tibia.

### 2.15 U2OS‐luc intracardiac inoculation xenograft model

As previously described,^40^ 4‐week‐old male nude mice were anesthetized with tribromoethanol. U2OS‐luc cells (1 × 10^6^) were resuspended in PBS and inoculated into their left ventricles using tuberculin syringes with 29‐gauge needles (BD bioscience). When the needle was implanted, blood jets were observed as a criterion for entering the left ventricle, and implantation was completed within 10 seconds. Three days later, Tan I (10 or 20 mg/kg/d) was intraperitoneally administrated to the mice for 32 days. On days 0, 12 and 24 post‐Tan I treatment, tumour metastasis was monitored using bioluminescence imaging after injection of D‐luciferin (Gold Biotechnology). Imaging data were analysed with the Living Image Software (Calliper Life Sciences). Survival curves of mice were also recorded.

### 2.16 Statistical analysis

Data were presented as means ± SEM, and statistical analysis was performed using Microsoft Excel and GraphPad Prism 5 software with the Student's *t* test. All experiments were repeated at least three times. *P* < .05 was considered statistically significant.

## 3 RESULTS

### 3.1 Tan I inhibits OS cell proliferation and colony formation

**Figure 1:**
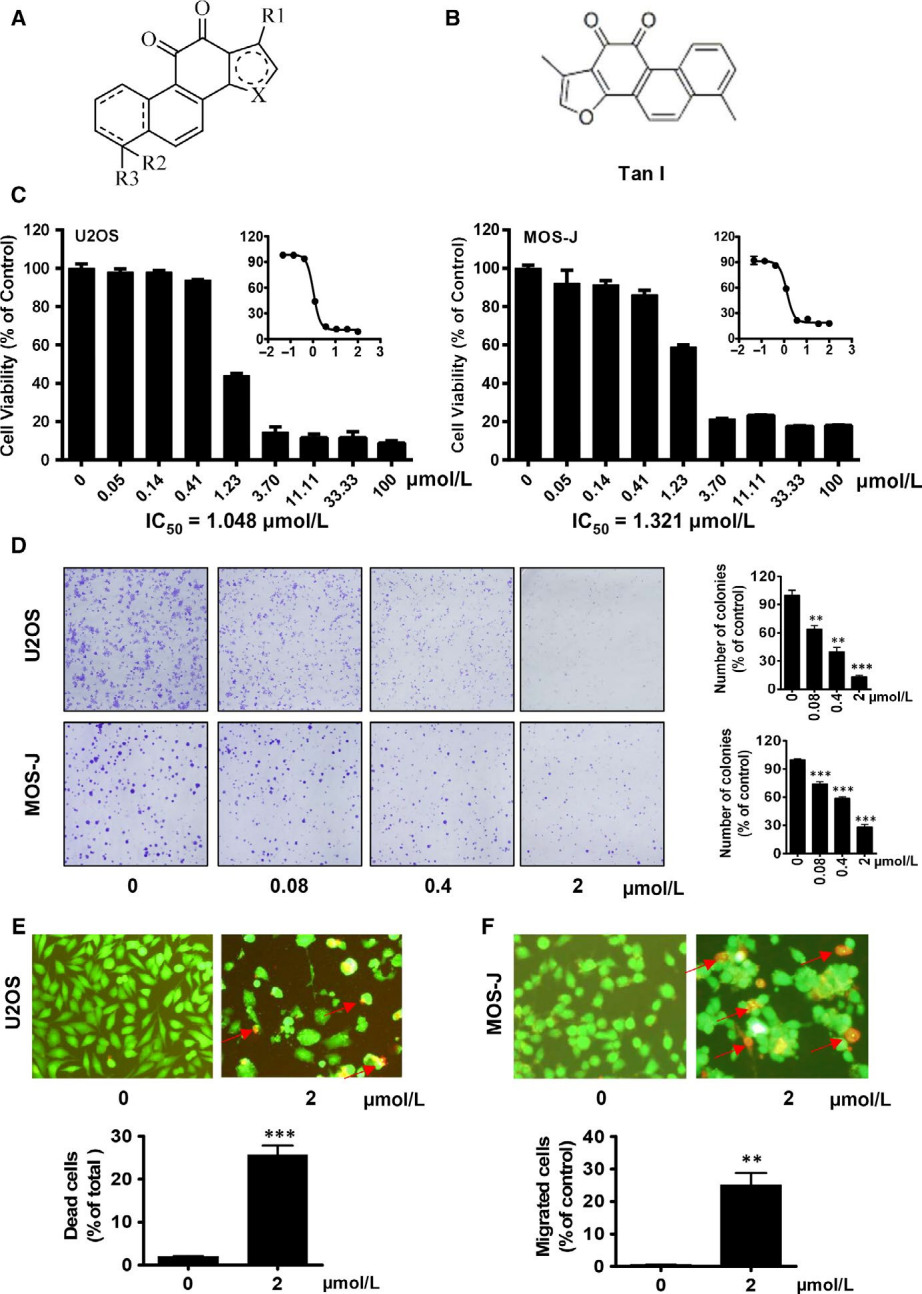
Tan I suppresses osteosarcoma cell proliferation in vitro. A and B, the general molecular formulas of tanshinone (A) and Tan I (B). C, Tan I suppresses U2OS and MOS‐J cell proliferation. Cells were treated with the indicated concentrations of Tan I for 72 h, and cell viability was assessed using the sulforhodamine B (SRB) assay . D, Tan I inhibited U2OS and MOS‐J cell colony formation. After treatment with the indicated concentrations of Tan I in 6‐well plates for a week, cells were fixed and stained with crystal violet, and cell colonies were counted. Quantitative data of the ratio of dead cells are shown at the right. E and F, Tan I destroyed U2OS (E) and MOS‐J cell (F) plasma membrane integrity. Cells were treated with the indicated concentrations of Tan I for 24 h and stained with the live‐dead kit. Dead and live cells were stained red and green, respectively. Quantitative data of the ratio of dead cells are shown at the bottom. **P < .01 and ***P < .001

As shown in Figure 1A,B, the chemical structure of Tan I is similar to that of tanshinones, derived from Danshen. To investigate the effects of Tan I on tumour proliferation, we first performed sulforhodamine B (SRB) assay in OS cells, including U2OS and MOS‐J cell line. We found that Tan I dose‐dependently inhibited U2OS and MOS‐J cell proliferation (Figure 1C). Tumour cell viability declined from 90% to 20% when the Tan I concentration was increased from 0.41 to 100 μmol/L, and its half‐maximal inhibitory concentration (IC 50) was about 1 μmol/L in both OS cancer cell lines. Next, cell colony formation assays to validate the effects of Tan I on U2OS and MOS‐J cell growth were performed. The results were consistent with those of the cell proliferation assay, as Tan I deceased colony formation in a dose‐dependent manner, and significantly inhibited cell viability at 0.4 μmol/L, compared with that in the control group (Figure 1D). The anti‐proliferative effect of Tan I was also confirmed using the live/dead assay. As shown in Figure1E,F, 2 μmol/L of Tan I significantly increased the proportion of dead cells in the U2OS and MOS‐J cell lines. These results demonstrate that Tan I can effectively inhibit OS cell growth in vitro.

### 3.2 Tan I inhibits OS cell migration and invasion

**Figure 2:**
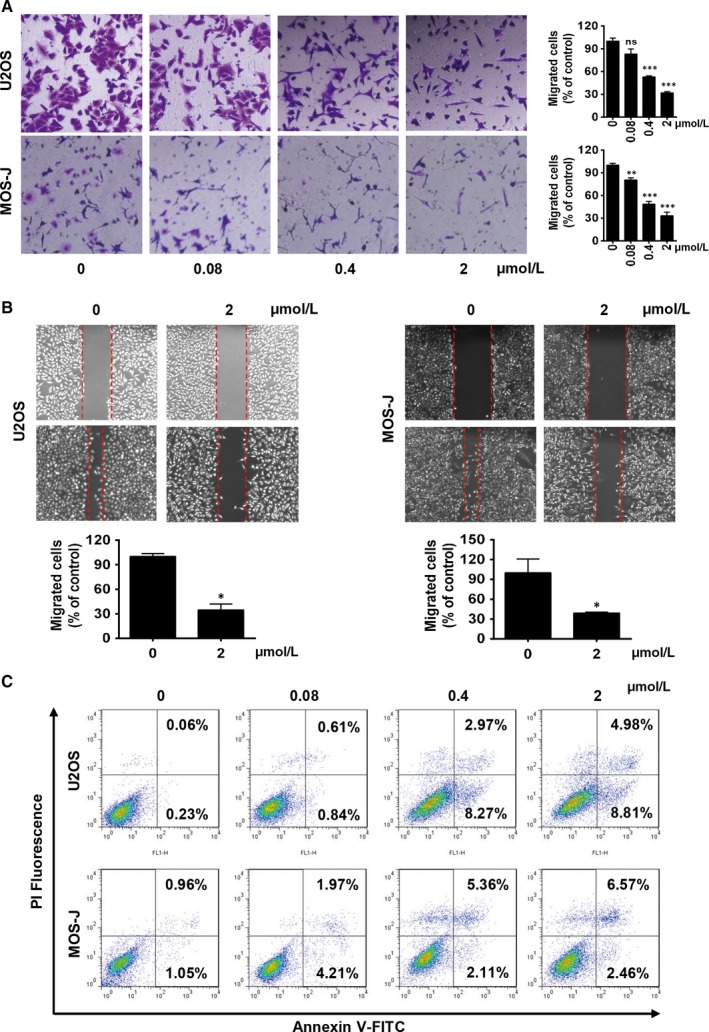
Tan Ⅰ inhibits the migration and induces the apoptosis of osteosarcoma cells in vitro. A and B, Tan I inhibits U2OS and MOS‐J cell migration. Cells were seeded into the top chamber and treated with the indicated concentrations of Tan I. Migrated cells were stained and quantified after 24 h (A). Cells were seeded in 6‐well plates and wounded using sterile pipette tip. After incubation for 24 h with the indicated concentrations of Tan I, the cells were fixed and photographed (B). C, Tan I mediated U2OS and MOS‐J cell apoptosis in a concentration‐dependent manner. Cells were treated with the indicated concentrations of Tan I for 48 h, and the percentage of apoptotic cells was analysed with flow cytometry and staining with PI and Annexin V‐FITC. Ns, no significant difference, *P < .05, **P < .01 and ***P < .001

Because of the association between OS cell migration, and invasion and metastatic potential, we conducted a chamber migration assay and a wound‐healing migration assay, to determine the effects of Tan I on OS cell migration and invasion. A cell migration assay by Boyden inserts showed that Tan I dose‐dependently inhibited U2OS and MOS‐J cell migrations, and that this inhibition was significant at 0.4 μmol/L of Tan I (Figure 2A). Similarly, Tan I significantly decreased U2OS and MOS‐J cell invasive properties (Figure 2B). As Tan I was found to induce U2OS and MOS‐J cell death, we examined the level of apoptosis by Annexin V and propidium iodide (PI) staining. We found that Annexin V and PI‐positive cells increased with increasing Tan 1 concentrations (Figure 2C). Therefore, Tan I could effectively suppress OS cell growth in vitro by inhibiting their proliferation and enhancing apoptosis.

### 3.3 Tan I inhibits U2OS subcutaneous tumour growth in vivo

**Figure 3:**
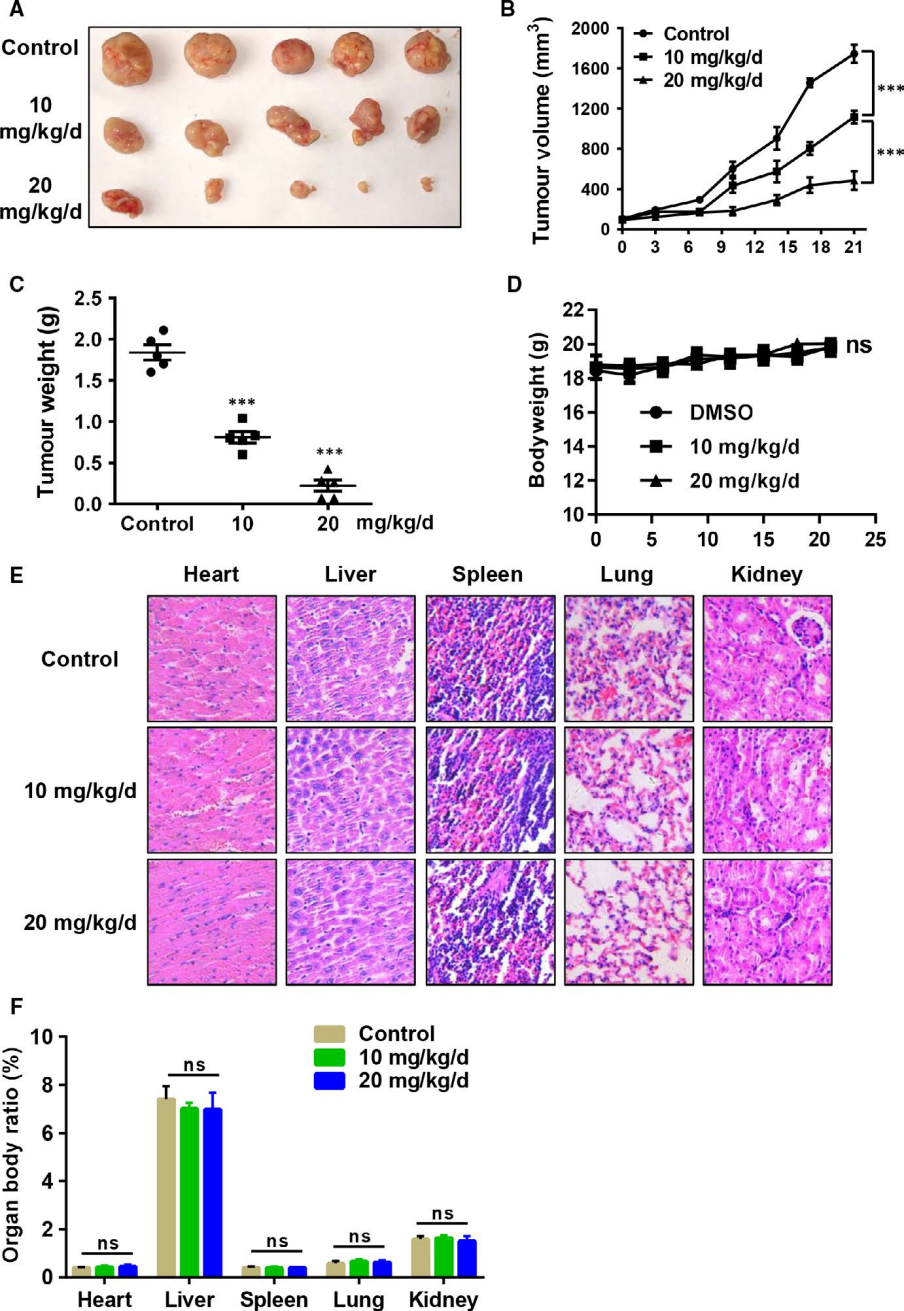
Tan I inhibits osteosarcoma cell growth in vivo. A, B, C and D, U2OS tumour‐bearing mice were intraperitoneally treated with 10 or 20 mg/kg Tan Ⅰ or vehicle daily. Tumour volume (B) and mice bodyweight (D) were measured twice a week during the administration period. The mice were killed after 21 d, and their tumours were removed (A) and weighed (C). E, H&E stained liver, spleen, kidney, heart and lung. F, Organ/body mass ratios after the mice were killed. Ns, no significant difference and ***P < .001

Given that Tan I can inhibit OS cell proliferation and colony formation and induce apoptosis in vitro, we wondered whether Tan I could inhibit OS growth in vivo. U2OS cells were injected into nude mice to establish the OS subcutaneous tumour xenograft model. The mice were then treated with 10 or 20 mg/kg Tan I or vehicle control for 21 days, killed, and their tumour xenografts were dissected (Figure 3A). As shown in Figure 3B and 3, Tan I notably suppressed OS tumour growth. The average tumour volume of the control group was 1746 ± 197 mm^3^, whereas that of the Tan I‐treated groups were 1116 ± 140 mm^3^ for the 10 mg/kg/d group and 484 ± 203 mm^3^ for the 20 mg/kg/d group. Moreover, the average tumour weight of the control group was 1838 ± 207 mg, while that of the Tan I‐treated groups were 808 ± 157 mg for the 10 mg/kg/d group and 220 ± 150 mg for the 20 mg/kg/d group. Besides, Tan I treatment at the given concentration had little effect on the bodyweight, compared with those of mice in the control group (Figure 3D). Pathological staining and organ/body ratio measurements showed no Tan 1‐induced toxicity in the hearts, livers, spleens, lungs and kidneys of the treated mice, compared with those of the mice of in the control mice (Figure 3E‐F). These results therefore indicate that Tan I exerts potent anti‐tumour efficacy in the U2OS tumour xenografts without obvious side effects.

### 3.4 Tan I inhibits orthotopic tumour growth and metastasis in vivo

**Figure 4:**
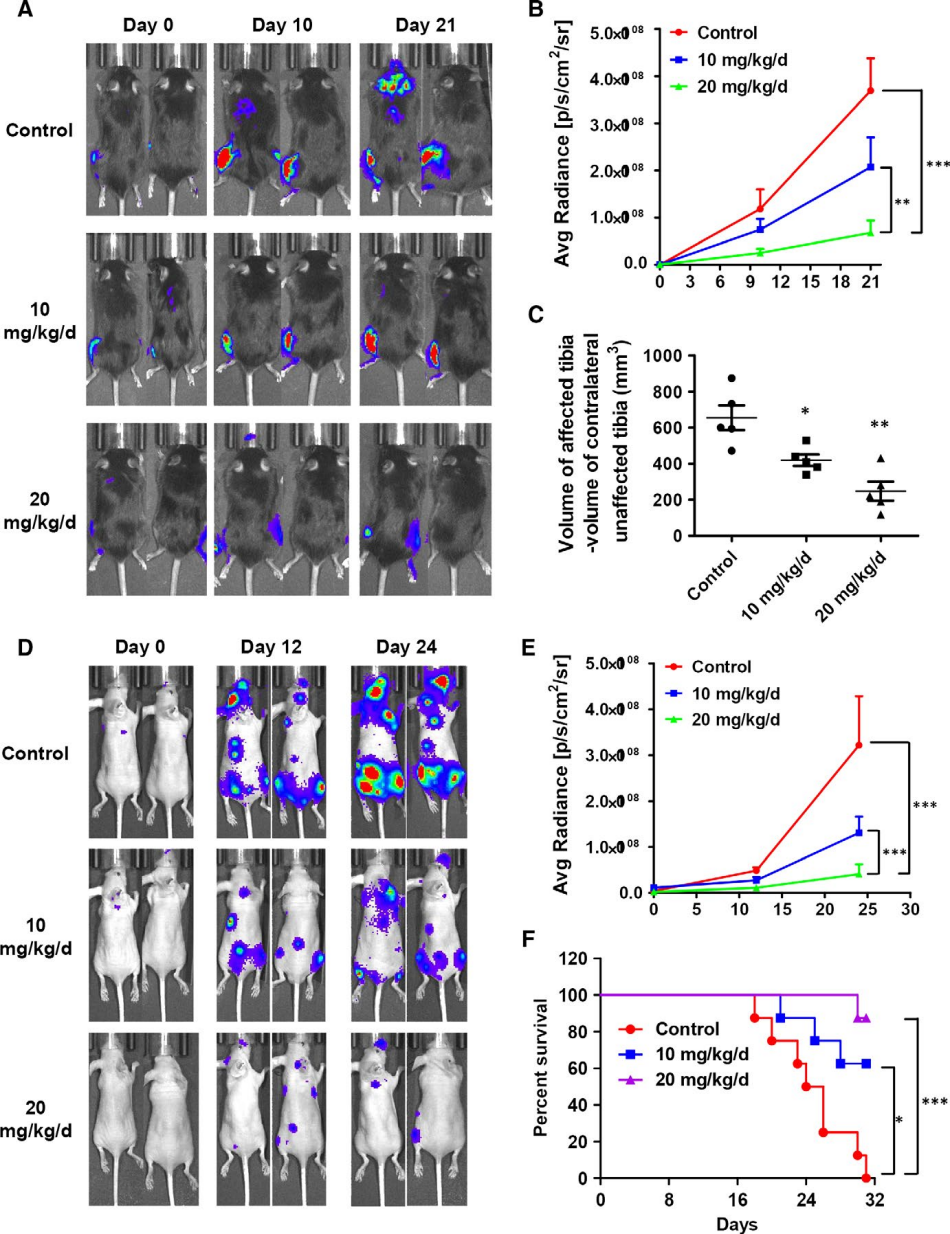
Tan I inhibits osteosarcoma cell metastasis in vivo. A, OS‐1‐luc cells were injected into the tibia of C57 mice, and the resulting tumour‐bearing mice were treated with 10 or 20 mg/kg Tan I or vehicle daily. The OS‐1‐luc tumours were imaged using IVIS on treatment days 0, 10 and 20. B, Tumour volume was determined using the normalized photon flux. C, Tumour length and width were measured using a digital calliper after harvest, and tumour volume was calculated with the equation volume = length × width^2^ × 0.52. D, U2OS‐luc cells were injected through the left ventricle, and the resulting tumour‐bearing mice were treated with 10 or 20 mg/kg Tan I or vehicle daily. U2OS‐luc tumours were imaged using IVIS on treatment days 0, 12 and 24. E, Tumour cell distribution was quantified using the normalized photon flux. F, Mouse survival curves during the treatment period. *P < .05, **P < .01 and ***P < .001

In an effort to closely mimic human disease, especially for confirming whether Tan I could regress OS in vivo, we evaluated Tan I's potential on the OS‐1 orthotopic tumour growth and metastasis model in vivo. For the orthotopic tumour model, a luciferase‐expressing OS‐1‐luc cell line (OS‐1‐luc) was established and intratibially injected into C57 mice. Following orthotopic injection, the mice were treated with 10 or 20 mg/kg Tan I for 21 days, and bioluminescence imaging was conducted to trace the OS‐1‐luc cells in vivo. As shown in Figure 4A‐C, Tan I treatment obviously regressed the OS‐1‐luc orthotopic xenografts in the tumour‐burdened mice. Daily treatment with 10 mg/kg Tan I significantly reduced the photon flux indexes, whereas daily treatment with 20 mg/kg Tan I almost completely blocked tumour growth (Figure 4A,B). Furthermore, daily treatment with 10 and 20 mg/kg Tan I significantly reduced tumour volume by 20% and 50%, respectively (Figure 4C).

Using a tumour metastasis model, we investigated the effect of Tan I on OS metastasis in vivo. U2OS‐luc cells were inoculated into the left ventricle of nude mice. Then, the mice were administered 10 or 20 mg/kg Tan I daily for 24 days, bioluminescence imaging was conducted, and the survival of mice was recorded. We found that both 10 and 20 mg/kg Tan I effectively inhibited tumour metastasis, as tumour metastasis was found in the control group mice (Figure 4D‐E). Moreover, Tan I treatment significantly increased mouse survival rates by 80% in the 20 mg/kg group and 60% in the 10 mg/kg group (Figure 4F). These results confirm that Tan I efficaciously suppresses the growth and metastasis of OS in vivo.

### 3.5 Tan I altered the expression of mRNAs and proteins involved in apoptosis and invasion

**Figure 5:**
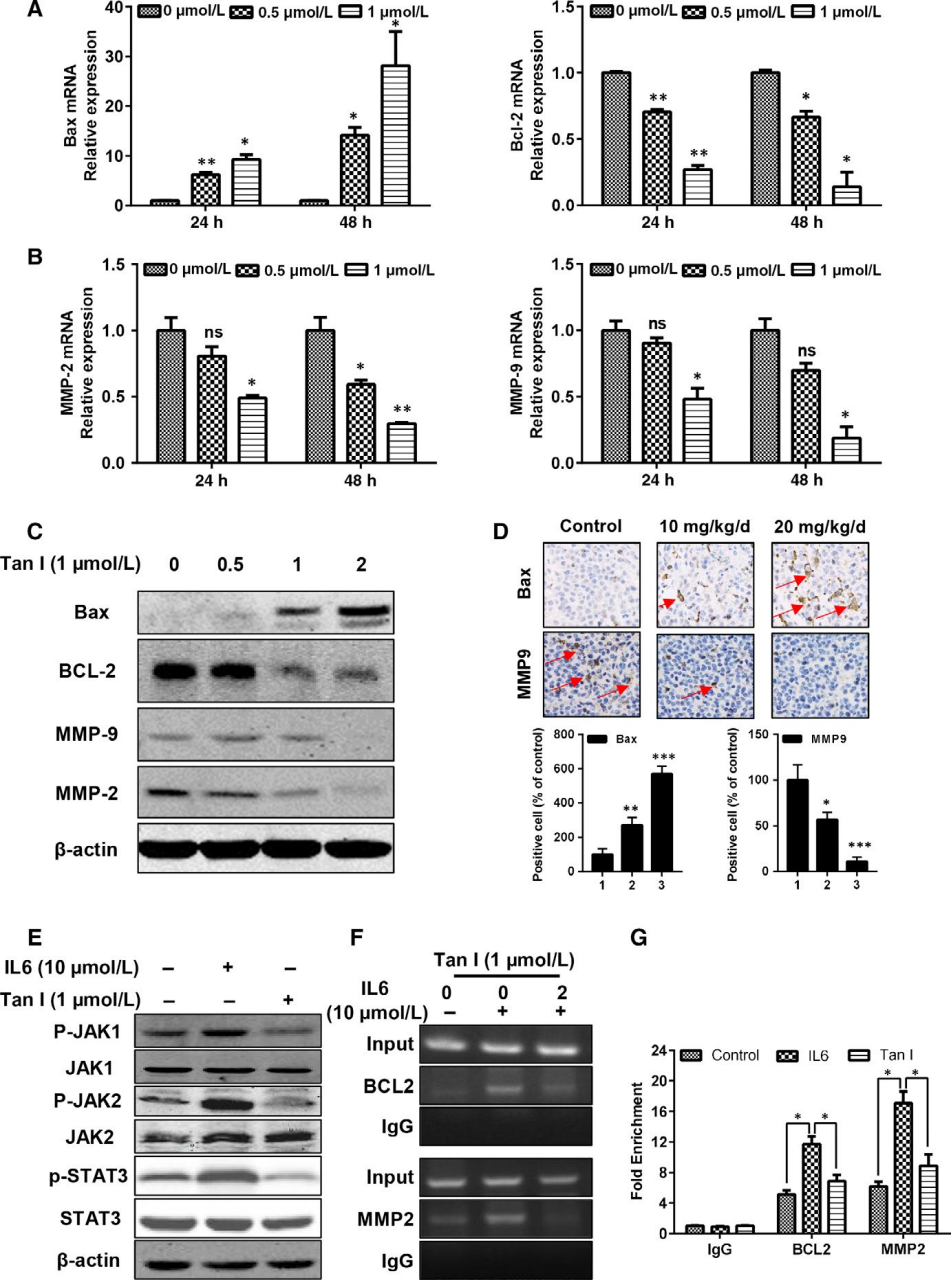
Tan I inhibits osteosarcoma growth and metastasis via JAK/STAT3 signalling pathway inactivation. A and B, The qPCR detected relative gene expression of Bax, Bcl‐2, MMP‐2 and MMP‐9, 24 or 48 h post‐Tan I treatment. C, Western blotting determined relative protein expression of Bax, Bcl‐2, MMP‐2 and MMP‐9 24 h post‐Tan I treatment. D, IHC staining of Bax and MMP9 in U2OS cells, Bax‐ and MMP9‐positive cells were indicated by red arrows. E, Tan Ⅰ suppressed IL‐6‐induced phosphorylation of JAK1, JAK2 and STAT3. F and G, U2OS cells were incubated with Tan I and analysed using a quantitative ChIP assay with anti‐STAT3 antibody. Ns, no significant difference, *P < .05, **P < .01 and ***P < .001

To clarify the anti‐tumour mechanism of Tan I, we determined cell apoptosis and invasion‐induced gene expression alterations, as Tan I‐induced cell apoptosis and significantly inhibited cell migration and invasion. We first examined the mRNA and protein expression of apoptotic molecules belonging to the Bcl‐2 family. We found that Tan I increased both the mRNA and protein expression of Bax and decreased those of Bcl‐2, in a dose‐dependent manner (Figure 5A,C). In addition, metalloproteinases (MMPs), also found in tanshinone isotypes such as Tan IIA, reportedly have a central role in extracellular matrix degradation to support OS invasion and metastasis.^21^ Therefore, we validated the mRNA and protein expression of MMP‐2 and MMP9 in Tan I‐treated U2OS cells. As expected, Tan I treatment markedly decreased MMP‐2 and MMP‐9 levels in a dose‐dependent manner (Figure 5B,C). Also, in evaluating Bax and MMP9 expression in U2OS cells post‐Tan I treatment in vivo, we found that treatment with 10 or 20 mg/kg Tan I significantly increased Bax expression, but drastically inhibited that of MMP9 (Figure 5D).

Epidermal growth factor (EGF) signalling is well known to support the migration and invasion of OS cells, as well as those of many other cancers,^41^ which we determined by examining the downstream JAK1/2‐STAT3 signalling pathway.^42^ As shown in Figure 5E, IL‐6‐induced phosphorylated JAK1, JAK2 and STAT3 up‐regulation activated the JAK1/2‐STAT3 signalling pathway. However, Tan I ameliorated this effect by decreasing the levels of phosphorylated JAK1, JAK2 and STAT3, indicating that Tan I can effectively block the activation of EGF‐induced downstream signalling. Moreover, ChIP assays revealed that Tan I inhibited the interactions between STAT3 and its target genes Bcl2 and MMP2 (Figure 5F‐G). These results show that Tan I inhibits OS cell growth and metastasis by targeting the JAK1/2‐STAT3 signalling pathway and exerting a proapoptotic effect.

## 4 DISCUSSION

Danshen, the traditional Chinese herb, and its derivatives are widely used to treat heart diseases and have no significant adverse effects.^43^, ^44^ Along with 30 diterpene compounds in Danshen, cryptotanshinone, tanshinone I and tanshinone IIA are the most abundant and well‐studied,^14^ and their anti‐cancer effects have been widely demonstrated.^9^, ^45^ In the present study, Tan I significantly inhibited the proliferation, migration and invasion of osteosarcoma (OS) cells in vitro, as well as tumour growth and metastasis in vivo. To the best of our knowledge, this is the first study to elucidate the OS cell growth inhibitory effects of Tan I.

Proliferation, migration and invasion are well known major features of tumour formation. We found that Tan I inhibited the proliferation of the OS cell lines U2OS and MOS‐J in a time‐ and dose‐dependent manner. Also, Tan I strongly decreased cell viability after 72 hours of treatment, and its IC50 was 1‐1.5 μmol/L (Figure 1C,D), much lower than those previously observed in other cancer cells.^26^, ^29^ In addition, Tan I exerted a powerful effect on cell migration and invasion. The chamber migration assay showed that treatment with 0.4 and 2 μmol/L of Tan I inhibited the migration of about 50% and 70% of OS cells, respectively (Figure 2A,B).

The induction of apoptosis is considered a crucial anti‐tumour mechanism of drugs.^46^, ^47^ Similar to the results of studies on colon cancer cells and HepG2 hepatoma cells, 48, 49 we found that Tan I significantly increased tumour cell apoptosis in a dose‐dependent manner (Figures 1E,F and 2C). We clarified the molecular mechanisms of Tan I's apoptosis‐inducing effect by determining the mRNA and protein expression of anti‐apoptotic and proapoptotic molecules such as Bcl‐2 and Bax, respectively. Our results indicated that Tan I induced cell apoptosis via Bax activation and Bcl‐2 suppression (Figure 5A,C).

Despite the abundant evidence of Tan I's anti‐cancer effects against various cancer cell lines in vitro, only a couple of reports suggest its anti‐cancer effects in vivo (lung, breast, gastric and prostate cancer).^25^, ^26^, ^28^, ^33^ We therefore investigated the in vivo effects of Tan I using both subcutaneous and orthotopic tumour growth models. Daily administration of 20 mg/kg Tan I for 21 days strongly inhibited tumour growth in vivo without obvious adverse effects (Figures 3 and 4A‐C). Because local or distant recurrences in OS patients involve approximately 90% lung metastases with very poor prognosis,^2^ it is vital to develop novel efficacious metastasis inhibiting drugs, especially those against OS. Tan I was reported to significantly inhibit lung tumour vascularity and metastasis,^33^ which we investigated using a metastasis model, and found that Tan I decreased tumour metastasis and significantly increased survival time of mice (Figure 4D,F). These results are exciting because the effects of tanshinones on tumour metastases have rarely been reported, although tanshinone IIA has been widely shown to inhibit OS cell growth in vitro and in vivo.^21^, ^50^ We therefore concluded that Tan I can effectively suppress OS growth and metastasis in mice.

Tumour‐associated proteases are required for metastasis, invasion and migration.^51^ Metalloproteinases (MMPs) are believed to be essential for extracellular matrix degradation, a significant characteristic of OS invasion and metastasis.^52^ In the present study, Tan I suppressed the mRNA and protein expression of MMP‐2 and MMP‐9, possibly responsible for OS cell invasion and metastasis (Figure 5B,C). Inflammatory cytokines and signalling pathways are well known to play pivotal roles in enhancing tumour metastasis and drug resistance.^53^, ^54^ IL‐6 secreted in the tumour microenvironment activates the JAK/STAT3 signalling pathway, favouring tumour growth and metastasis.^55^, ^56^ In determining the potential mechanism, we found that Tan I blocked IL‐6‐induced JAK1/2 and STAT3 activation and down‐regulated p‐JAK1/2 and STAT3 protein levels (Figure 5E‐G). It is worth mentioning that a number of oncogenic signalling pathways are involved in OS progression,^47^ and we could not exclude the association between Tan I treatment and the other signalling pathways. From these findings, it could be assumed that Tan I inhibits the JAK1/2‐STAT3 signalling pathway and suppresses MMP‐2 and MMP‐9 expression, thereby blocking OS invasion and metastasis in mice.

This study had several limitations. Firstly, although Tan I inhibited OS cell proliferation, the detailed molecular mechanism such as regulation of cell cycle or microtubule dynamics, remained unclear.^57^ In addition, the mechanism of Tan I‐induced OS cell apoptosis and death remained unclear.^58^ Besides, the drug metabolism, pharmacokinetics and pharmacodynamics of Tan I and other potential mechanisms involved in its anti‐tumour in vivo effects needed further elucidation.

In summary, Tan I was efficacious in suppressing OS cell growth and metastasis both in vitro and in vivo. Tan I induced cell apoptosis by activating Bax and inhibited tumour proliferation and metastasis by inactivating the JAK‐STAT3 signalling pathway. Therefore, Tan I could be considered and further investigated as a novel, efficient and safe drug candidate for the treatment of OS progression.

## CONFLICT OF INTEREST

The authors declare no conflict of interest.

## AUTHOR CONTRIBUTIONS

W Wang and J Li designed the research; W Wang, J Li, Z Ding and Y Li performed the experiments; J Wang and S Chen analysed the data; J Li and S Chen wrote the manuscript; J Miao edited the manuscript; J Li obtained the funding and supervised the work with J Miao. All authors read and approved the final manuscript.

## Data Availability

The data that support the findings of this study are available from the corresponding author upon reasonable request.
